# In vitro evaluation of the potential role of sulfite radical in morphine-associated histamine release

**DOI:** 10.1186/1471-2210-4-21

**Published:** 2004-10-06

**Authors:** Emma M Gordon, Carolyn Myers, Jeffrey Blumer

**Affiliations:** 1 Division of Pediatric Pharmacology and Critical Care, Department of Pediatrics, Case Western Reserve University, Cleveland, Ohio, USA

## Abstract

**Background:**

Intravenous morphine use is associated with elevated histamine release leading to bronchoconstriction, edema and hemodynamic instability in some patients. This study evaluated the possibility that sulfite, which is present as a preservative in many morphine preparations, might contribute to histamine release in vitro.

**Results:**

The human mast cell line, HMC-1, was exposed to various morphine concentrations, in the absence of sulfite, under cell culture conditions. Clinically attained concentrations of morphine (0.018μg/ml and 0.45μg/ml) did not cause increased histamine release from mast cells. There was a significant increase in histamine release when the morphine concentration was increased by 1184-fold (668μg/ml morphine). Histamine release from mast cells exposed to morphine and/or sulfite required the presence of prostaglandin H synthetase. Histamine release in experiments using sulfite-containing morphine solutions was not statistically different from that observed in morphine-only solutions.

**Conclusion:**

Sulfite in sulfite-containing morphine solutions, at concentrations seen clinically, is not responsible for histamine release in *in vitro *experiments of the human mast cell line, HMC-1. This does not preclude the fact that sulfite may lead to elevation of histamine levels *in vivo*.

## Background

Morphine is among the most common analgesic agents used in the intensive care setting. It is usually administered parenterally as an intravenous infusion. Despite its efficacy, its use is associated with a number of hazards including respiratory depression [1], tolerance [2], physiological dependence [2] and abstinence upon discontinuation of treatment [2]. Less frequent, but perhaps more profound, is the bronchoconstriction, edema and hemodynamic instability seen in some patients following intravenous bolus doses of the drug [3-7]. These effects have been attributed to histamine release from mast cells by morphine [3-7].

A number of clinical studies have demonstrated an increase in plasma histamine concentration following intravenous morphine administration [3-7], but these studies generally fail to provide details concerning the formulation of the morphine employed. Most of the morphine used in both clinical practice and for clinical investigations contains sodium bisulfite as a preservative [8]. Until the mid 1980's sulfite preservatives were generally considered universally safe; however, their status was changed after the receipt by the FDA of more than 250 reports of serious and life-threatening reactions related to sulfite exposure through both diet and therapeutic agents [9,10].

In the body, sulfites are generally oxidized by the mitochondrial enzyme sulfite oxidase and non-toxic metabolites are excreted in the urine [11]. A small amount of the sulfite, either ingested or injected may also be metabolically activated by a number of other enzyme systems including xanthine oxidase [12], peroxidase [11] and prostaglandin H synthetase [13]. As an electron acceptor in these reactions or through electron transfer from transition metals such as copper and iron, sulfite may accept an electron from the resulting hydroxyl, superoxide or sulfate radicals [14] leading to the formation of sulfite radical.

Sulfite radicals are chemically reactive and have been implicated in lipid [15], protein [16] and nucleic acid [17] oxidation as well as neuronal injury [18]. They may stimulate neutrophils to release oxygen free radicals and augment the free radical response to other neutrophil activators [19]. In addition, their generation has been shown to contribute to oxidative stress resulting in sulfite toxicity and impaired B-cell function.

The potential generation of free radicals from sulfite preservatives may confound our understanding the association of morphine with histamine release in man. A number of studies have demonstrated that, when exposed to free radicals, mast cells may release histamine [20-26]. It is possible; therefore, that the histamine released following morphine infusion may be the result of the formation of sulfite radicals from the preservative rather than the morphine itself. This study evaluated the possibility that sulfite and its activation might contribute to histamine release by mast cells in vitro.

## Results

### Mast cell histamine release

Total histamine content of mast cells was calculated by freezing and thawing suspensions for 3 cycles. Cell suspensions containing 500,000 cells/ml released 1.27μg/ml (0.21) histamine, corresponding to 2.53 pg (0.41) histamine per mast cell.

Mast cells stimulated with 0.25μg/ml calcium ionophore released 63% (7.2) of total histamine.

### Effect of sulfite-free morphine concentration on histamine release

The effect of histamine release from mast cells treated with both clinically attained concentrations of morphine and concentrations above those seen clinically was determined.

To chemically defined reaction mixtures, morphine was added at varying concentrations (18 ng/ml, 450 ng/ml, 6.68μg/ml and 668μg/ml). Enzymes causing free radical activation were not present. The blank sample contained no morphine. Reaction mixtures were incubated in a water bath at 37°C for 60 minutes. Experiments were performed in triplicate. Histamine release from samples was determined following derivitization with o-phthaldialdehyde and mercaptoethanol using high-performance liquid chromatography as detailed below. Histamine release from reaction mixtures was expressed as a % of total histamine present in the cell pellet.

Histamine release from reaction mixtures containing morphine at all concentrations was not statistically different (p > 0.1) from histamine release from blank samples (Table 1).

**Table 1:** Histamine release from HMC-1 cells stimulated with varying concentrations of morphine with and without sulfite Histamine release expressed as a percentage of release of histamine in comparison to control (SD). As a control, the HMC-1 cells were allowed to freeze and thaw for 3 cycles to release the remaining histamine from cells. Each value represents the mean (SD) of triplicate determinations.

**Morphine concentration (μg/ml)**	Histamine release (% total)* Sodium bisulfite	
	+	-
0		2.9 (2.6)
0.018	2.5 (0.75)	3.2 (1.1)
0.450	1.7 (0.4)	2.6 (0.4)
6.68	1.8 (0.6)	2.2 (1.3)
668	2.1 (0.06)	2.8 (0.2)

**Figure 1:**
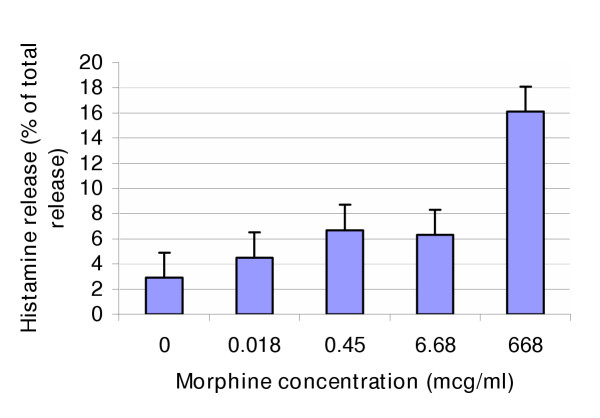
Concentration-effect of morphine in the presence of prostaglandin Hsynthetase on histamine release from the mast cell line HMC-1 (mean of 3 experiments).

To verify the time dependence of histamine release, a morphine concentration of 668 μg/ml was tested. Release was detectable as early as 5 minutes after the addition of drug and appeared to plateau between 40 and 60 minutes (Figure 1).

### Effect of sulfite-containing morphine solutions on histamine release

To determine whether the addition of sodium bisulfite causes increased histamine release from mast cells, sodium bisulfite at a concentration of 0.1% of morphine concentration (the % of sulfite present in morphine most formulations) was added to the reaction mixtures described previously. Blank samples contained neither morphine nor sulfite. Specimens were incubated, dried, re-dissolved, derivitized and analyzed as above.

There was no statistical difference in histamine release at any concentration of morphine with sulfite compared with blank samples (Table 1).

Reaction mixtures containing 668μg/ml morphine with 0.1% sodium bisulfite were incubated for 5, 10, 20, 40 and 60 minutes yielded results identical to those observed in the absence of sodium bisulfite (results not shown).

### Effect of prostaglandin H synthetase on histamine release from mast cell reaction mixtures

Prostaglandin H synthestase may catalyze free radical formation [20]. The first experiment investigated its effect on mast cell histamine release in the absence of sulfite and morphine. This ensured that any increase in histamine release in subsequent experiments was not due to the effect of the enzyme only.

Prostaglandin H synthetase was added to reaction mixtures at a concentration of 25 mU. In reaction mixtures containing no drug, the enzyme was added to mast cell samples containing neither morphine nor sulfite. Controls contained no enzyme.

Reaction mixtures containing the enzyme alone (no sulfite or morphine) produced similar histamine release compared to blank samples (3.2% (0.7) of total and 2.9% (2.6) of total respectively). The presence of prostaglandin H synthetase had no effect.

The effect of clinically attained concentrations of morphine in the presence of prostaglandin H synthetase on mast cell histamine release was investigated. *In vivo *studies show histamine release at these concentrations of morphine [3-7]. Morphine sulfate was added to the mast cell solution at clinically attained concentrations of 450 ng/ml (a typical peak *in vivo *concentration following an intravenous morphine bolus) and 18 ng/ml (a typical steady-state concentration during a 20 μg/kg/hour morphine infusion) [27]. Mast cell histamine release from higher concentrations of morphine (668μg/ml and 6.68μg/ml) was also investigated.

Blank samples contained water instead of morphine. All samples contained 25 mU of prostaglandin H synthetase. Specimens were incubated, dried, re-dissolved, derivitized and analyzed as described below. Histamine release values are shown in Table 2. There was no statistical difference between histamine release from morphine samples at both clinically-attained concentrations (18 ng/ml (p = 0.87) and 450 ng/ml (p = 0.08)) and histamine release from blank samples. Histamine release from HMC-1 cells incubated with morphine 668μg/ml and 6.68μg/ml were significantly increased compared with blank samples (p = 0.03 at both concentrations). Results are shown in table 2. Maximum histamine release (16.1% of total (6.3)) occurred at the highest concentration of morphine (668μg/ml) (Figure 1).

**Table 2:** Histamine release from HMC-1 cells stimulated with varying concentrations of morphine with and without sulfite in the presence of prostaglandin H synthetase. Histamine release expressed as a percentage of release of histamine in comparison to control (SD). As a control, the HMC-1 cells were allowed to freeze and thaw for 3 cycles to release the remaining histamine from cells. Each value represents the mean (SD) of triplicate determinations.

**Morphine concentration (μg/ml)**	Histamine release (% total)* Sodium bisulfite	
	+	-
0		2.9 (2.6)
0.018	3.3 (2.9)	4.5 (1.4)
0.450	6.5 (0.9)	6.7 (1.8)
6.68	8 (0.3)	6.2 (1.5)
668	17.6 (7.8)	16.1 (6.3)

The effect of sodium bisulfite 0.1% in varying concentrations of morphine in the presence of prostaglandin H synthetase was investigated. This preservative is present in many morphine preparations and its effect on histamine release from mast cells is determined here. Morphine was added to the mast cell samples at concentrations of 668μg/ml, 6.68μg/ml, 450 ng/ml and 18 ng/ml. Sodium bisulfite was added to samples at a concentration of 0.1% of morphine concentration. Controls contained morphine only. Each sample also contained 25 mU of prostaglandin H synthetase. Specimens were incubated, dried, re-dissolved, derivitized and analyzed as described below. Reaction mixtures containing morphine 668μg/ml with 0.1% sodium bisulfite showed a statistically significant increase in histamine release compared with blank samples (p = 0.03). There was no statistical difference in histamine release between morphine only samples at all concentrations and samples containing morphine and 0.1% sodium bisulfite (Table 2) (morphine 668μg/ml (p = 0.8), morphine 6.68μg/ml (p = 0.13), morphine 450 ng/ml (p = 0.89) and morphine 18 ng/ml (p = 0.56). Sulfite did not have any additive effect on histamine release from mast cells in the presence of morphine and prostaglandin H synthetase.

Studies looking at plasma histamine levels following morphine administration in humans have shown peak histamine levels within 5 minutes of administration [3,4,6]. This experiment investigated whether maximum histamine release occurred at a similar time *in vitro *in the presence of the enzyme prostaglandin H synthetase. HMC-1 cells were incubated at 37°C for 5, 10, 20, 30 and 60 minutes with morphine 668μg/ml, 0.1% sodium bisulfite and 25 mU prostaglandin H synthetase. The cells were centrifuged and the supernatant was examined for the presence of histamine and expressed as a % of total histamine.

**Figure 2:**
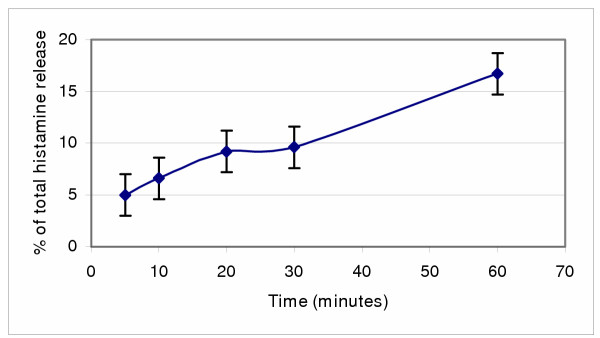
Time response effects of morphine 668 μg/ml in the presence of 0.1% Na bisulfite and 25 mU prostaglandin H synthetase on histamine release in HMC-1 cells (mean of 3 experiments).

Histamine release was time dependent with a maximum effect after 1 hour of incubation (Figure 2).

Interestingly, reaction mixtures containing a typical peak morphine concentration (450 ng/ml) showed a significant increase in histamine release in the presence of prostaglandin H synthetase compared with no enzyme. This was true for both sulfite-free (p = 0.003) and sulfite-containing (p = 0.009) morphine solutions. This suggests that even at clinically-attained morphine concentrations, it is not only possible to cause the metabolic activation of sulfite, but also that of morphine.

The effect of sulfite alone (without morphine) on histamine release from mast cells was investigated. To 4 mast cell reaction mixtures, varying concentrations of sulfite (0.018 ng/ml, 0.45 ng/ml, 0.007μg/ml and 0.67μg/ml) were added. These concentrations of sulfite correspond to the concentration of sulfite present in morphine solutions of 18 ng/ml, 450 ng/ml, 6.6μg/ml and 668μg/ml. To each sample 25 mU prostaglandin H synthetase was added. The highest concentration of sulfite (0.67μg/ml) was incubated for 10, 20, 40 and 60 minutes to assess time dependence. The reaction mixtures were incubated, centrifuged and analyzed as described below.

In the presence of prostaglandin H synthetase, sulfite resulted in histamine release from mast cells. There was a significant increase in histamine release (9.7% (0.65) of total) in the reaction mixture containing 0.67μg/ml sulfite (p = 0.01). There was no statistically significant difference in histamine release compared with blank samples at all other concentrations of sulfite (0.018 ng/ml sulfite (2.8% (0.3) of total, p = 0.91), 0.45 ng/ml sulfite (1.93% (0.35) of total, p = 0.53) and 0.007μg/ml sulfite (4.5% (0.7) of total, p = 0.36) (Table 3).

**Table 3:** Histamine release from HMC-1 cells stimulated with varying concentrations of sulfite-only solutions in the presence of prostaglandin H synthetase. Histamine release expressed as a percentage of release of histamine in comparison to control (SD). As a control, the HMC-1 cells were allowed to freeze and thaw for 3 cycles to release the remaining histamine from cells. Each value represents the mean (SD) of triplicate determinations.

**Sulfite concentration (ng/ml)**	**Histamine release (% total)**
0.018	2.8 (0.3)
0.45	1.93 (0.35)
**7**	4.5 (0.7)
670	9.7 (0.65)

The time-dependent experiment showed histamine release of 8% of total (3.3) at 10 minutes incubation, 7.7% of total (2.2) at 20 minutes, 10.5% of total (6.1) at 40 minutes and 9.7% of total (0.65) at 60 minutes.

## Discussion

Our experiments showed similar histamine release from the mast cell line HMC-1 stimulated with morphine with or without 0.1% sodium bisulfite. Maximum histamine release was 16.1% of total in morphine only samples and 17.6% of total in morphine with 0.1% sodium bisulfite samples. This percentage is similar to other studies of histamine release in the human mast cell line HMC-1, where maximum release was 20–30% [28-30]. In our study, the concentrations of morphine and sulfite required to achieve maximum histamine release were significantly higher than concentrations seen clinically. Reaction mixtures containing sulfite only also caused significant histamine release at high concentrations. Blank samples, containing neither morphine nor sulfite, did not cause a significant increase in histamine release (Table 2). These results suggest that at high concentrations, both sulfite and morphine could be responsible for elevated histamine release from mast cells. In addition to the formation of the sulfite radical from sulfite, it is possible that carbon-centered or nitrogen-centered free radicals are being generated from the metabolic activation of morphine [20], thereby inducing mast cell histamine release.

HMC-1 cells contain histamine and release their histamine content in response to various exogenous stimuli [28-30]. Our study showed no evidence of elevated histamine release from mast cells when stimulated with morphine and/or sulfite at concentrations seen clinically (both peak and steady-state). Although HMC-1 exhibits a phenotype similar to that of human mast cells [31], it is possible that immature cells (cell lines) are less sensitive than mature (primary) cells [28]. The model used is an artificial system and may not represent physiological conditions. In addition, maximum histamine release occurred after a one hour incubation. This is significantly different from the *in vivo *situation, where maximum release usually occurs within 5 minutes of morphine administration, and suggests that we were not able to simulate exactly the human environment where histamine release occurs. It is likely that although the sulfite radical is formed rapidly *in vitro*, it may take some time for its effects to become evident [32].

Metabolic activation of sodium bisulfite may occur through a variety of mechanisms. We investigated a number of enzyme systems and our final model used prostaglandin H synthetase. Other enzyme systems (horseradish peroxidase/ hydrogen peroxide and hypoxanthine/ xanthine oxidase) did release histamine in our model (results not shown), but they produced excess background interference in the chromatograms and made identification of peaks difficult. Activation of sulfite following morphine administration may not occur through this mechanism, however, and is another possible flaw in our aim to construct a model similar to the one that exists *in vivo*.

Morphine has been shown to cause significant histamine release *in vivo, *but it has not been investigated whether sulfite, in the form of the sulfite radical, is the cause of this release. Our study finds that sulfite in sulfite-containing morphine solutions, at clinically attained concentrations, is not responsible for histamine release in *in vitro *experiments of the mast cell line HMC-1, but this does not preclude the fact that sulfite may lead to marked elevation in histamine levels *in vivo*. The release of a significant amount of histamine with either sulfite or morphine at concentrations higher than those seen clinically suggests that both substances may be responsible for histamine release from mast cells. Further clinical research studies need to be performed to examine this possibility.

## Conclusions

Our study finds that the sulfite in sulfite-containing morphine solutions, at concentrations seen clinically is not responsible for histamine release in *in vitro *experiments of the mast cell line, HMC-1, but this does not preclude the fact that sulfite may lead to marked elevation in histamine levels *in vivo*. Our experiments suggest that at high concentrations, both morphine and sulfite are responsible for histamine release from mast cells. Further *in vivo *research needs to be performed to examine this possibility.

## Methods

### Preparation of HMC-1 cells

Human leukemic mast cells, HMC-1 [33] were kindly provided by J. Butterfield, Rochester, Minnesota. This cell line shows many characteristics of immature mast cells. They have been shown to release histamine following stimulation [28-30] Cells were carried in 75 cm^2 ^vented tissue culture flasks (BD Falcon, Franklin Lakes, NJ) containing Iscoves modified Dulbecco's medium (Sigma, St.Louis, MO) with 10% iron-supplemented calf serum (Hyclone, Logan, Utah) and monothioglycerol 1.2 mM (Sigma, St.Louis, MO). Incubations were conducted in a humidified atmosphere at 5% CO_2 _and 37°C. Cells were passed weekly and fed only when necessary (evidence of color change in media).

To pass cells, the cell suspension was placed in a 50 ml polypropylene centrifuge tube (BD Falcon, Franklin Lakes, NJ) and centrifuged at 250 × g at 4°C for 10 minutes. The culture medium was discarded and the cells were re-suspended in fresh medium. Cell density was maintained at 500,000 cells/ml.

### Chemicals

Reagent grade O-phthaldialdehyde (OPA), mercaptoethanol, dihydrogen sodium phosphate, disodium EDTA, sodium bisulfite, morphine sulfate, hydrogen peroxide, xanthine oxidase, boric acid and phosphate buffered saline (PBS) were purchased from Sigma (St.Louis, MO, USA). Methanol (HPLC grade), acetonitrile (HPLC grade) and sodium hydroxide were from Fisher Scientific (Hampton, NH). Phosphate buffered saline (PBS) pH 7.4, contained 120 mmol/L NaCl, 2.7 mmol/L KCl and 10 mmol/L phosphate. Horseradish peroxidase was purchased from Cooper Biomedical. Prostaglandin H synthetase was purchased from Calbiochem (Darmstadt, Germany).

### Determination of histamine release

Mast cell suspensions, containing approximately 500,000 mast cells were, aliquoted into 1 ml samples. These were rinsed 4 times with 4 ml of PBS, pH 7.4. Cells were then re-suspended in 1 ml PBS. Reaction mixtures were supplemented with morphine, sulfite and prostaglandin H synthase at this stage according to experimental design, unless otherwise specified. Reaction mixtures were incubated for 60 minutes in a water bath at 37°C. Reactions were stopped by centrifugation at 250 × g for 4 minutes at 4°C and placed on ice. Then 200μl of the supernatant was collected and dried under nitrogen at 40°C.

Histamine in the supernatant was measured using derivitization with OPA and mercaptoethanol (ME) as described [34]. Dried samples were re-dissolved in 100μl of derivitisation buffer (0.4 M boric acid adjusted to pH 9.5 with 1 M sodium hydroxide) and then derivitized with 20 μL of a freshly made 1:1 mixture of OPA (3.8 mM in methanol) and ME (2.5 ml/L in methanol) and analyzed within 30 minutes. The derivitized samples were used for chromatography.

The response of HMC-1 cells to the stimulant calcium ionophore was investigated to assess the releasability of HMC-1 cells. 0.25μg/ml calcium ionophore was added to mast cell suspensions. Reaction mixtures were incubated and analyzed as above.

### Recovery of total histamine from cells

To extract the remaining histamine from the original sample, the mast cell pellet in suspension was frozen and thawed 3 times. It was then centrifuged at 250 × g for 4 minutes and 200μl of the supernatant was dried under nitrogen as described previously. Samples were re-dissolved, derivitized and analyzed as above. Histamine release during morphine incubations was expressed as a percentage of total histamine release.

### Chromatography system

Histamine was measured by high-performance liquid chromatography using a Varian model 5500 chromatograph. The mobile phase was 0.1 M dihydrogen sodium phosphate and 1 mM disodium EDTA with 16% methanol and 14% acetonitrile adjusted to pH 6.4. The flow rate was 0.8 ml/minute.

Separations were performed using a 15 cm × 4.6 mm, Ace 5 C18 reversed-phase cartridge column (Advanced Chromatography technologies, UK). A Zorbax C8 guard column with a 5 micron, 4.6 × 12.5 mm analytical guard-column cartridge (Agilent technologies, Palo Alto, CA) was used. Fluorescence detection was used with the fluorimeter (Varian 9070) set at an excitation of 348 nm and an emission of 444 nm (determined by running individual excitation and emission spectra on the fluorometer).

Chromatographic conditions involved the use of a mobile phase gradient to optimize peak separation. A linear gradient starting at 90% mobile phase, 10% acetonitrile and ending at 80% mobile phase, 20% acetonitrile took place over 20 minutes. The column was washed by increasing the acetonitrile to 30% by 22 minutes and by 23 minutes the proportions were returned to the original values. The length of each run was 30 minutes.

### Data analysis

Values are expressed as a mean +/- SD of 3 experiments. One-way analysis of variance was used to determine the significance of the difference between groups. Results were considered significantly different when p < 0.05.

## Authors' contributions

EG participated in experiments and drafted the manuscript. JB conceived of the study and participated in its design. CM suggested the methodology of experiments. All authors read and approved the final manuscript.
